# Computational analysis of data from a genome-wide screening identifies new *PARP1* functional interactors as potential therapeutic targets

**DOI:** 10.18632/oncotarget.26812

**Published:** 2019-04-12

**Authors:** Samuele Lodovichi, Alberto Mercatanti, Tiziana Cervelli, Alvaro Galli

**Affiliations:** ^1^ Yeast Genetics and Genomics Group, Laboratory of Functional Genetics and Genomics, Institute of Clinical Physiology CNR, Pisa, Italy; ^2^ PhD Student in Clinical and Translational Science Program, University of Pisa, Pisa, Italy

**Keywords:** PARP1, genome wide screening, functional interactors, cancer therapy targets

## Abstract

Knowledge of interaction network between different proteins can be a useful tool in cancer therapy. To develop new therapeutic treatments, understanding how these proteins contribute to dysregulated cellular pathways is an important task. PARP1 inhibitors are drugs used in cancer therapy, in particular where DNA repair is defective. It is crucial to find new candidate interactors of PARP1 as new therapeutic targets in order to increase efficacy of PARP1 inhibitors and expand their clinical utility. By a yeast-based genome wide screening, we previously discovered 90 candidate deletion genes that suppress growth-inhibition phenotype conferred by PARP1 in yeast. Here, we performed an integrated and computational analysis to deeply study these genes. First, we identified which pathways these genes are involved in and putative relations with PARP1 through g:Profiler. Then, we studied mutation pattern and their relation to cancer by interrogating COSMIC and DisGeNET database; finally, we evaluated expression and alteration in several cancers with cBioPortal, and the interaction network with GeneMANIA. We identified 12 genes belonging to PARP1-related pathways. We decided to further validate RIT1, INCENP and PSTA1 in MCF7 breast cancer cells. We found that RIT1 and INCENP affected PARylation and PARP1 protein level more significantly in PARP1 inhibited cells. Furthermore, downregulation of RIT1, INCENP and PSAT1 affected olaparib sensitivity of MCF7 cells.

Our study identified candidate genes that could have an effect on PARP inhibition therapy. Moreover, we also confirm that yeast-based screenings could be very helpful to identify novel potential therapy factors.

## INTRODUCTION

Gene network analysis may reveal protein's interaction with functional and therapeutic significance that could lead to development of new therapeutic treatment with higher clinical utility. Moreover, integration data analysis could be a useful tool to improve efficiency of drugs when resistance develops, because it gives the opportunity to have more targets to treat with a combination approach and reduce frequency of resistance.

Here, we focused on the poly (ADP-ribose) polymerase 1 (PARP1) gene and its interaction network, because PARP1 inhibition is widely used in cancer therapy. Knowledge of its interactions could not only help to discover new applications of these inhibitors in different cancers with specific dysregulated pathways related to PARP1. PARP1 encodes for a nuclear protein that attaches a poly(ADP-ribose) polymer (PAR) to itself and other target proteins dealing with DNA repair and related pathways; PARylation is necessary to activate DNA repair and to bring proteins to the sites of damage and is reported to be stimulated in presence of DNA damage [1, 2]. Generation of PAR following stresses is an extremely rapid process; however, PAR is also catabolized rapidly by proteins that promote PAR degradation [3–5]. The prompt turnover of PAR is crucial for efficient DNA repair. Defects in PAR catabolism result in DNA damage that is deleterious to cells [6, 7]. Considering that cells are continuously exposed to various types of DNA-damage agents, DNA repair mechanisms must be robust and almost free of errors to ensure cell survival [8].

Repair of DNA single strand breaks (SSB) is the main role of PARP1; this kind of damage is repaired through different mechanisms such as base excision repair (BER) and nucleotide excision repair (NER). PARP1 has been reported to be involved in all these DNA repair pathways [9]. PARP1 is also involved in repair of DNA double strand breaks (DSB) that are the most severe type of DNA damage and must be repaired by mechanisms free of errors to preserve DNA sequence integrity. PARP1 has been shown to recognize DNA DSBs and recruits factors to facilitate DSB repair either by homologous recombination (HR) or non-homologous end joining (NHEJ) [10, 11]. In addition, PARP1 has a role in other cellular pathways like DNA replication, chromatin structure modification and cell cycle control. In particular, PARP1 seems to have a role in stabilization and restart of arrested replication fork, and can facilitate nucleosome disassembly by histone PARylation resulting in chromatin relaxation [12–17]. Moreover, by remaining stably associated with certain promoters, PARP1 can also activates genes involved in transcription restart after completion of mitosis [18].

Inhibition of PARP1 in cancer therapy is a strategy used in those types of cancers where DNA repair is defective causing an effect called “synthetic lethality”. By definition, a synthetic lethal interaction occurs between two genes when the perturbation of either gene alone is viable but the perturbation of both genes simultaneously results in cell death [19]. Thus, PARP1 inhibitors are mainly used in breast and ovary tumors carrying defects in the homologous recombination gene *BRCA1* or *BRCA2*, in combination with DNA damaging agents such as cisplatin or topoisomerase-1 inhibitors [20]. So far, olaparib, rucaparib and niraparib have been approved by the US Food and Drug Administration (FDA) [21, 22]. These inhibitors bind the catalytic domain of PARP1 leading to a reduction of PARylation and, therefore, to a defect in DNA repair [23, 24]. Moreover, some evidence suggests that PARP1 inhibitors also cause formation of PARP1-DNA complexes with increased cytotoxicity and cell death [25]. However, some patients develop resistance to PARP1 inhibitors, leading to treatment failure [26]. Resistance to PARP1 inhibitors is one big issue that needs to be solved to increase efficiency of drugs and chance of patient survival [27]. There are three principal mechanisms of resistance: restoration of *BRCA1/2* function through “reverse” mutations, partial restoration of HR through somatic loss of 53BP1, a NHEJ factor that when deleted, promotes damaged DNA ends to produce ssDNA ends competent for HR, and upregulation of multidrug efflux transporters such as P-glycoprotein (Pgp) resulting in a higher rate of drug efflux [28–30]. In order to overcome this limitation, it is of pivotal importance to identify proteins that modulate PARP1 activity that could be new target to increase efficiency of PARP inhibitors or to generate new drugs that could be used in combination with already existing ones. This combinational approach with PARP1 inhibitors has been already used with success in specific cancers and several clinical trials are ongoing [20]. For this reason, the identification of predictive biomarkers and the ability to overcome PARP1 inhibitor resistance will be crucial to enable further optimization of PARP1 inhibitors for cancer therapy; moreover, understanding PARP1 relation network could expand utility of PARP1 inhibitors also to other cancers where these interactors or related pathways are altered.

Recently, through a genome-wide genetic screening in the yeast *Saccharomyces cerevisiae,* we have identified 90 candidate genes potentially affecting PARP1 activity; in particular, when these genes are deleted in yeast, PARP1 cannot perform its activity and yeast cells are able to grow [31]. Here, we performed an integrating computational analysis of these candidate genes by interrogating several databases. Then, we carried out functional validation and we identified three genes that could have a role in PARP1 activity and could be considered new promising therapeutic targets.

## RESULTS

Previously, we reported that expression of *PARP1* is lethal in yeast. We have also demonstrated that *PARP1*-induced lethality is due to PARP1 activity [31]. Moreover, we have identified PARP1 putative new “functional interactors” that potentially could either directly interact with PARP1 or just be involved in related pathways and modulate PARP1 function. We found 90 gene deletions able to suppress the *PARP1*-induced lethality in yeast; this indicates that these genes are somehow involved in PARP1 activity [31]. For the sake of clarity and completeness, the list of these genes was reported and shown in Table 1. We thought it could be interesting to study more in depth these interactors, in order to find candidate proteins that could modulate PARP1 activity and to assess if these new putative interactions could be exploited to develop new therapeutic treatments.

**Table 1:** Genes identified with the yeast genome wide screening, and related pathways

Gene Name	Total number of genes	Related Pathway
CRNKL1, PAN2, RPUSD1, SART1, SNRPC, WBP11, ZCCHC7, ZFP36, ZFP36L1, ZFP36L2, NUP210, RRN3	12	mRNA processing and transport
GALK1, GALK2, GCK, HK1, HK2, HK3, HKDC1, ALG6, LDHD, MOGS, PSAT1, NADK, ENTPD5, ENTPD6, PIKFYVE, WDR85	16	Cellular metabolism
SLC2A6, SLC2A8, SLC30A1, SLC30A10, SLC30A2, SLC30A3, SLC30A4, SLC30A8, SLC35B4, SLC44A1, SLC44A2, SLC44A3, SLC44A4, SLC44A5, SLC7A13, SLC7A14, SLC7A4	17	Aminoacids and Sugar Transport
H1F0, H1FNT, H1FX, HIST1H1T, NCOR1, NCOR2, RCOR1, RCOR2, RCOR3, PRMT5	10	Chromatin Assembly
RAP1A, RAP1B, RAP2A, RAP2B, RAP2C, ZFYVE21, ZFYVE28	7	Cell proliferation and adhesion
INCENP, ZNF207	2	Chromosome segregation
HECTD2, HERC3, HERC4, HERC5, HERC6, UBE3A, YOD1	7	Ubiquitination and ubiquitin-related pathway
AGPAT3, AGPAT4, AGPAT5, AGPS, LCLAT1, TALDO1,	6	Phospholipids biosynthesis
RIT1, RIT2	2	MAPK pathway
REXO1, REXO1L1, REXO1L10P, REXO1L11P	4	Ribosome biogenesis
ARGLU1, HTATSF1, LENG8, SH3YL1, TCP11, TCP11L1, TCP11L2	7	Other Pathways

List of the genes is taken from La Ferla *et al*., 2015 [31]. Genes are grouped in pathways where they belong.

### Most genes belong to cellular metabolism, RNA processing and chromatin remodeling pathways

**Figure 1:**
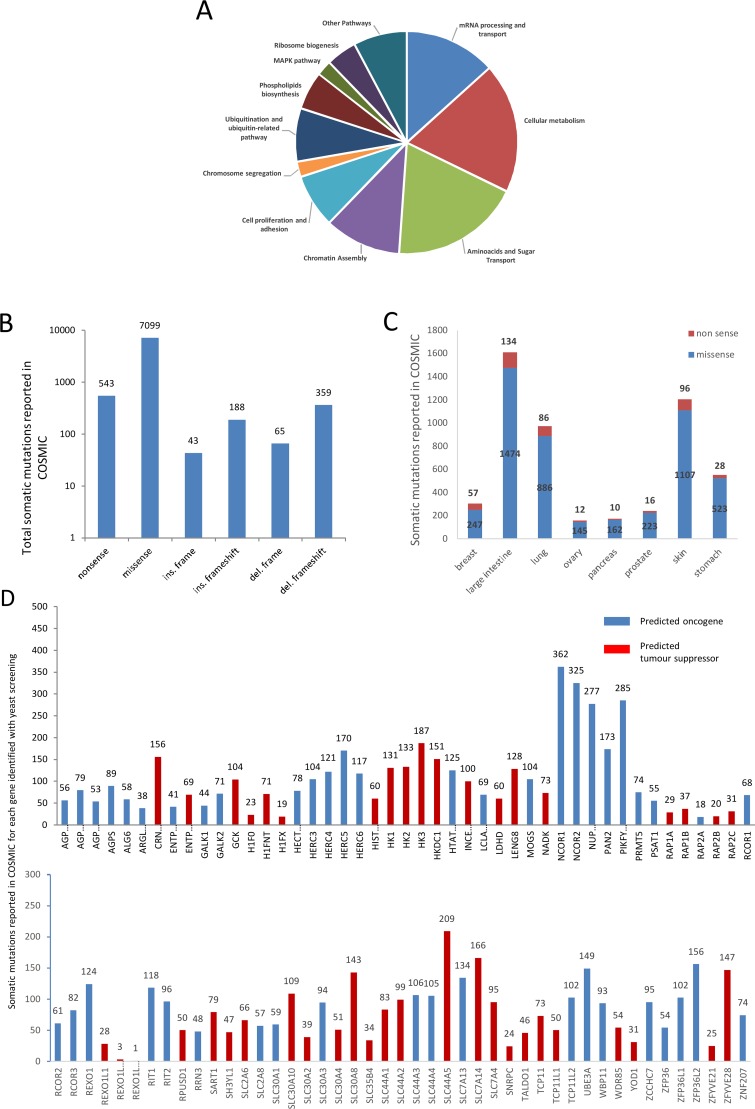
The genes identified by the yeast-based screening belong to several cellular pathways and are mutated in different kinds of cancer **(A)** “Cellular metabolism” and “amino-acid and sugar transportation” are the pathways where most genes belong. “mRNA processing and transport” and “Chromatin assembly” are pathways where PARP1 is deeply involved. Also, pathways such as “Chromosome segregation” and “MAPK” are related to PARP1. **(B)** Total mutations are retrieved from COSMIC. Data are grouped for type of mutation. The number of mutations is reported at the top of any histogram. **(C)** Number of mutations found in different cancers (from COSMIC) where PARP1 inhibition is currently used in therapy (breast, ovarian and prostate cancer) or where clinical trials are ongoing (intestine, lung, pancreas, melanoma and gastric cancer). Missense and nonsense mutations are shown for each kind of cancer. The number of nonsense and missense mutations of all the genes is reported for any kind of cancer. **(D)** all the genes are reported to carry somatic mutations in Cancer (COSMIC). Total number of somatic mutations for each gene is indicated. Genes are divided between probable “oncogene” and probable “tumor suppressor” applying 20/20 rule [44].

To gain more information about these genes/proteins, we performed a computational integration analysis by interrogating several databases to see whether these genes are mutated or differentially expressed in cancers. First, we manually divided these genes into cellular pathways where they are involved; from metabolism to epigenetic control: the total number of genes belonging to each pathway is shown in Figure 1A. As much as 37% of genes (33/90) belong to cellular metabolism and sugar transport. Defects in this type of pathways may cause energy metabolism reprogramming that leads to modification in NAD^+^ biochemistry and, finally, affects PARP1 activity. Nevertheless, these genes can be also considered as a positive control of our yeast assay because alterations in galactose uptake and metabolism could just led to defect in *PARP1* promoter induction. Moreover, 13% (12/90) and 11% (10/90) of the genes belong to mRNA processing/transport and chromatin assembly, respectively (Table 1, Figure 1A); this again confirms the reliability of our assay, since it is known that PARP1 is involved in these mechanisms [32, 33]. Out of 90 genes, 7 belong to ubiquitination and cell proliferation pathway, and 4 to ribosome biogenesis (Table 1, Figure 1A); 2 genes belong to MAPK and chromosome segregation pathway (Table 1, Figure 1A) that are related to PARP activity [34–37]. Finally, 6 out of 90 genes are part of the phospholipids biosynthesis pathway (Table 1, Figure 1A).

### Identified genes are reported to be mutated in cancer

Next, we looked for the type of mutations and which mutations are more frequent, and in which type of tumors these genes are more likely mutated. To investigate whether these genes have been reported to be mutated in different types of cancer, we have queried the COSMIC database; total number of somatic mutations is shown in Figure 1B. A total of 8,297 mutations belonging to all 90 genes are reported; missense mutations represent the 86% of total mutations. As expected, mutations such as: in frame insertion/deletion and complex type are less frequent than the other types (Figure 1B).

Then, we analyzed how those mutations are distributed in different cancers. As PARP1 inhibitors are used in clinical therapy of breast, ovarian and prostate cancer, and clinical trials are ongoing to analyze their efficacy in melanoma, colorectal, pancreatic and gastric cancer [38–41], we analyzed how somatic mutations of our candidate genes are distributed among these cancers (Figure 1C). These genes are reported to be mostly mutated in large intestine, lung, skin and stomach cancer (Figure 1C).

The distribution and the number of somatic mutations among candidate genes is very variable and ranges from just one single mutation (REXO1L11P) to 362 mutations (NCOR1) (Figure 1D). The chromatin remodeling complex genes NCOR1 and NCOR2 are the most frequently mutated genes; a high level of somatic mutations was also found for NUP210, a glycoprotein member of the nuclear pore membrane and PIKFYVE an enzyme involved in inositol phosphate metabolism which is required for endocytic-vacuolar pathway and nuclear migration [42, 43]. Applying the 20/20 rule [44], we were able to classify our genes in probable tumor-suppressors or oncogenes (Figure 1D, Supplementary Table 1).

### 12 genes out of 90 are associated to cancer

In order to understand the involvement of these genes in neoplastic occurrence, we interrogated the DisGeNET database and we identified 12 genes associated to neoplasms. All results were filtered for DisGeNET Score value greater than 0.2 (Table 2). More information about these genes, such as: gene alterations and related publications, retrieved from DisGeNET, can be found in the Supplementary Table 2. These 12 genes have been deeper analyzed with g:Profiler in order to find more specific interaction with PARP1 (Supplementary Table 3). As shown in Table 2, genes identified by this analysis belong to the following cellular pathways: chromatin remodeling (H1F0, NCOR1 and RCOR1), cell division (INCENP), mRNA processing (ZFP36, ZFP36L2), ribosome biogenesis (REXO1), MAPK pathway (RIT1) and serine biosynthesis (PSAT1). Moreover, this enriched functional analysis showed a statistically significant interaction of PARP1 with these genes in several cellular processes/components (Supplementary Table 3).

**Table 2:** List of 12 candidate genes identified though DisGeNet analysis

Gene Name	Cancer Type	Gene Description	Pathway
**ENTPD6**	Seminoma	ectonucleoside triphosphate diphosphohydrolase 6 (putative)	Extracellular nucleotides catabolism
**H1F0**	Leukemia, Myelocytic, Acute	H1 histone family member 0	Chromatin remodelling
**HERC3**	Liver neoplasms	HECT and RLD domain containing E3 ubiquitin protein ligase 3	Ubiquitination
**INCENP**	Melanoma	inner centromere protein	Cell division
**NCOR1**	Mammary Neoplasms	nuclear receptor corepressor 1	Chromatin remodelling
	Bladder Neoplasm		
	Carcinoma, Transitional Cell		
	Glioblastoma		
	Liver neoplasms		
**PSAT1**	Non-Small Cell Lung Carcinoma	phosphoserine aminotransferase 1	Serine biosynthesis
**RCOR1**	Colonic Neoplasms	REST corepressor 1	Chromatin remodelling
**REXO1**	Polycystic Ovary Syndrome	RNA exonuclease 1 homolog	Ribosome biogenesis
**RIT1**	Lymphangioma, Cystic	Ras like without CAAX 1	MAPK regulation
**TALDO1**	Liver carcinoma	transaldolase 1	Lipid biosyntehsis
**ZFP36**	Neoplasm Invasiveness	ZFP36 ring finger protein	mRNA degradation
**ZFP36L2**	Prostatic Neoplasms	ZFP36 ring finger protein like 2	

All genes are related to cancer and have alterations or variants reported in database. All candidate genes have been analyzed and their relation with cancer has been calculated with DisGeneNet database which gives a score of this association from 0 to 1 based on type of sources (level of curation, model organisms) and the number of publications supporting the association. We selected genes that have a score higher than 0.2.

In addition, to query for drug-gene interactions, we interrogated DGIdb, and we found that 2 gene products have drugs acting among pathways where they are involved (Table 3): notably, selumetinib is already reported that to have a higher effect when used in combination with PARP inhibitors [45]. Therefore, this suggests that modulating the activity of these gene products it could improve efficacy of PARP1 inhibitors.

**Table 3:** Gene products that are already targeted by drugs

Gene Name	Drug Name	Alternative name	Target	Pathway	Source
INCENP	N~6~-CYCLOHEXYL-N~2~-(4-MORPHOLIN-4-YLPHENYL)-9H-PURINE-2,6-DIAMINE	Reversine	Aurora kinase	Cell division	DrugBank
RIT1	GDC-0941	Pictilisib	PI3K	MAPK	CIViC
RIT1	AZD-6244	Selumetinib	MEK	MAPK	CIViC

Drug-gene information has been retrieved from DGIdb as described in the materials and methods. Name and alternative name of drugs are showed; moreover, the actual target, pathway where target belong, and data-sources is showed.

### cBioPortal analysis identified interactions at different levels in cancers

**Figure 2:**
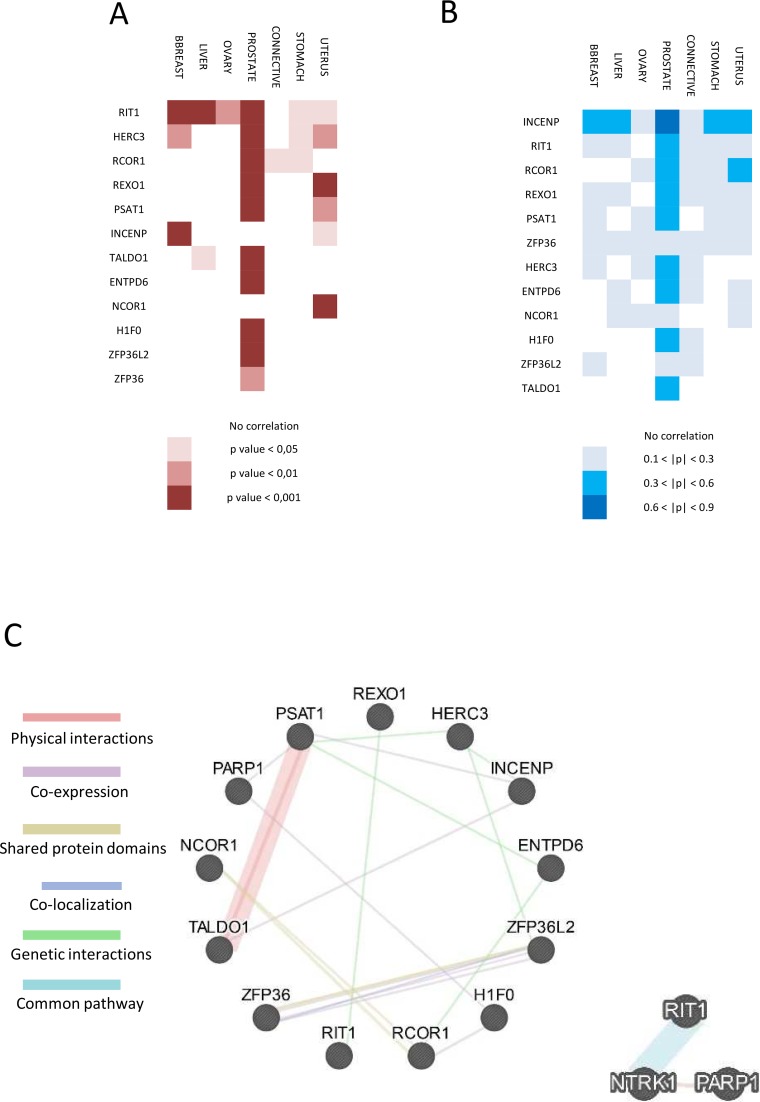
Functional interaction and correlation between PARP1 and the 12 selected genes **(A)** Heat-map of amplification correlation between PARP1 and the selected 12 genes in cancer from cBioPortal. Lowest *p*-value means highest correlation and most frequent de-regulation of their pathway in cancer. **(B)** Heat-map of expression correlation between PARP1 and the 12 genes in cancer samples from cBioPortal. Higher Pearson coefficient (p) means high correlation indicating that PARP1 and the selected gene are highly expressed in that kind of cancer/tissue. **(C)** GeneMANIA network between the selected proteins and PARP1 (left). Physical and genetic interactions, co-expression, shared protein domains co-localization and common pathway are shown by different colors. On the right, network between RIT1 and PARP1 is shown.

We analyzed the 12 DisGeNET-identified genes in selected human cancers with cBioPortal in order to identify any correlation between gene expression and PARP1 in selected human cancers. Any kind of interaction, between these genes and PARP1 could suggest an impact also in PARP1 activity. Based on DisGeNET results, we performed this analysis in 8 different cancer data for a total of 2,466 samples and 2,176 patients as showed in Table 4. In these cancer data sets, these genes are altered in 30–55% of samples/patients (Table 4). We first performed a co-occurrence /mutually exclusivity analysis in order to see if alterations such as; gene amplification, deletion or mutation in PARP1, statistically co-occur or are mutually exclusive with the same kind of alterations in the candidate genes. Results of this analysis are showed in Figure 2A. In all cancer samples there is association between PARP1 alteration, and, at least, one gene; in all the cases this association is a co-occurrence of amplification; this co-occurrence is particularly frequent for RIT1 that was found to be amplified in 6 out of 8 cancer types, suggesting a genetic interaction between their pathways and a possible common de-regulation in cancer (Figure 2A). Interestingly, in the case of neuroendocrine prostate cancer, alteration in all the genes with the exception of NCOR1, showed a statistically significant co-occurrence with PARP1.

**Table 4:** The selected genes are found to be altered in defined cancer studies

Cancer type	Data set size	% Genes alteration
Pancreatic Cancer (UTSW, Nat Commun 2015)	109 patients, 109 samples	55 %
Uterine Corpus Endometrial Carcinoma (TCGA, PanCancer Atlas)	509 patients / 509 samples	40 %
Ovarian Serous Cystadenocarcinoma (TCGA)	316 patients, 316 samples	40 %
Neuroendocrine Prostate Cancer (Trento/Cornell/Broad 2016)	77 patients, 107 samples	46 %
Breast Invasive Carcinoma (TCGA)	963 patients, 963 samples	30 %
Liver Hepatocellular Carcinoma (TCGA)	373 patients, 373 samples	31 %
Stomach Adenocarcinoma (TCGA, Nature 2014)	287 patients, 287 samples	33 %
Sarcoma (TCGA)	252 patients, 254 samples	35 %

The selected genes retrieved from DisGeNet analysis have been analyzed with cBioPortal. We reported data referred to cancer samples where the selected genes were found altered in at least 30% of cases. The size of data set and the percentage of gene alterations are shown.

Next, we performed a co-expression analysis to address whether PARP1 expression is correlated to expression of candidate genes in different cancers. These data are expressed as Pearson correlation coefficient (p) and are shown in Figure 2B. Interestingly, 9 out of 12 genes showed a medium expression correlation with PARP1 in prostate cancer; INCENP showed the highest correlation (p > 0.6). Moreover, INCENP revealed a medium correlation with PARP1 also in breast, liver, ovarian and stomach cancer (Figure 2B). All the data and coefficient values are present in Supplementary Table 4.

### GeneMANIA interaction network

Results obtained with DisGeNET were supported by GeneMANIA database. We confirmed some of the previous predicted interaction identifying co-expression of PARP1 with H1F0 and PSAT1. Moreover, we identified a physical interaction between TALDO1 and PSAT1 (Figure 2C. left). We then analyzed the interaction network between PARP1 and RIT1 or INCENP by enlarging the network also to identify new interactors. No additional INCENP interactor was found; interestingly, results obtained for RIT1 strengthened interaction to PARP1, since NTRK1 has been identified as a common member in their network (Figure 2C, right).

### Expression atlas analysis identifies specific differential expression

**Figure 3:**
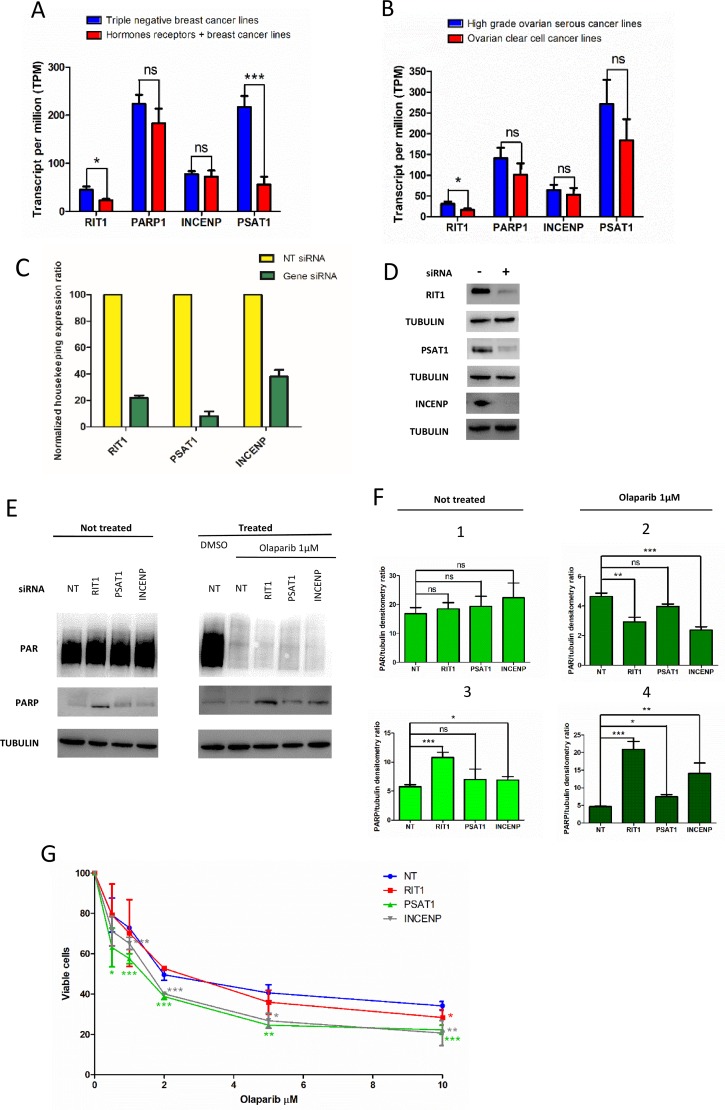
Effect of RIT1, INCENP and PSAT1 on PARylation and PARP1 protein level, and olaparip sensitivity **(A)** Expression of INCENP, RIT1 and PSAT1 in triple negative and hormone receptor positive (+) breast cancer cell lines: **(B)** Expression of INCENP, RIT1 and PSAT1 in high grade serous and ovarian clear cell cancer lines. Data were compared and statically analyzed by Student's *t*-test as indicated: ns, not significant, ^*^p<0.05, ^***^p<0.001. **(C)** Expression level measured by qRT-PCR of total RNA extracted from siRNA-transfected cells. Results are the mean of 3-4 experiments ± SD. **(D)** Western blot analysis of total protein extracts from MCF7 cells transfected with siRNA. Each lane was loaded with 30μg of protein as reported in Materials and Methods. Primary and secondary antibodies are described in the Materials and Methods. Tubulin was evaluated as loading control. **(E)** PARylation and PARP1 level in cell transfect with siRNA and treated with the PARP inhibitor “olaparib”. Western blot analysis of total protein extracts from siRNA-transfected MCF7 cells to measure PARylation and PARP1 level in olaparib not-treated (left) and treated (right) cells. Each lane was loaded with 30μg of protein as reported in Materials and Methods. Antibodies are described in the Materials and Methods. Tubulin was evaluated as loading control. **(F)** Densitometry was carried out by direct normalization on tubulin level. In panels 1 and 2, data on PARylation level are shown; in panels 3 and 4, data on PARP1 protein level. Panels 1 and 3 are referred to not-treated cells, panels 2 and 4 to olaparib-treated cells. Results are the mean of 3-4 experiments ± SD. Statistical analysis was performed using the Student's *t*-test; ns, not significant, ^*^p< 0.05, ^**^p< 0.01, ^***^p< 0.001. **(G)** Effect on RIT1, INCENP and PSAT1 on olaparib sensitivity in MCF7 cells. Cells are transfected with specific siRNA and after 6 hours re-plated in presence of different doses of olaparib (0, 5, 1, 2, 5, 10 μM) or DMSO. After 48 hours cell viability is calculated as described in Materials and Methods. Results are reported as mean of 3-4 experiments ± SD. Statistical analysis was performed using the Student's *t*-test by comparing data from siRNA-not-transfected (NT) to siRNA-transfected cells; ^*^p< 0.05, ^**^p< 0.01, ^***^p< 0.001.

To validate the data from this computational analysis, we decided first to check the mRNA expression level of RIT1, INCENP and PSAT1 the “EMBL-EBI expression atlas” database. We selected hormone receptors positive (+) and triple negative breast cancer cell lines (Figure 3A), and high grade ovarian serous and ovarian clear cell lines (Figure 3B). We compared the expression in cell lines from PARP1 inhibitor treatable cancers (triple negative breast and high grade serous cancers) with cell lines from PARP1 inhibitor not-treatable cancers (hormone receptors-breast cancer and ovarian clear cell cancer) (see Materials and Methods). Results showed that RIT1 expression was higher (p<0.05) in triple negative breast cancer and high grade ovarian cell lines than in the other cell lines; PSAT1 expression is significantly higher in triple negative breast cancer cell lines (p<0.001) as compared to the other breast cell lines. Expression of PARP1 and INCENP showed no statistically significant difference (Figure 3A and 3B).

### PARylation analysis and drug sensitivity

To evaluate influence on PARP1 activity, we down-regulated the expression of RIT1, PSAT1 and INCENP by using siRNA and analyzed the level of PARylation in MCF7 breast cancer cell line. We performed these experiments in the presence of the PARP inhibitor “olaparib” to assess if down regulation of these genes could affect PARylation and have an additive effect with olaparib treatment.

After assessing efficiency of selected siRNAs by qRT-PCR (Figure 3C) and by western blot analysis (Figure 3D), we measured PAR and PARP1 level by western blot analysis in MCF7 cell lines treated with olaparib and specific siRNA (Figure 3E, right and left panel). Densitometry showed that down-regulation of RIT1 and INCENP determined a statically significant decrease in PAR level only when olaparib is added (Figure 3F, panel 1 and 2). No effect was seen without drug treatment (Figure 3F, panel 2). Moreover, a statistically significant increase in PARP1 level was seen when RIT1 is down-regulated (Figure 3F, panel 3). When olaparib was added, PARP1 level significantly increased in RIT1- and INCENP-siRNA treated cells (Figure 3F, panel 4). Finally, we determined the olaparib sensitivity in MCF7 cells after inhibiting RIT1, INCENP or PSAT1 by specific siRNA (Figure 3G). Cells with low level of INCENP and PSAT1 were more sensitive to olaparib at almost any concentration tested. Precisely, PSAT1-inhibited cells showed a statistically significant reduction in cell survival at any olaparip concentration; olaparib significantly reduced survival in INCENP-inhibited cells at concentration higher than 1μM. In RIT1 inhibited cells, olaparib statistically reduced survival at the highest dose (10μM) (Figure 3G).

## DISCUSSION

As PARP1 inhibitors could affect several DNA repair pathways, they are widely used in cancer therapy because this treatment could lead to cancer cell death through a mechanism called “synthetic lethality”[19]. One drawback of cancer treatment with PARP1 inhibitors is the onset of resistance that often occurs. This means that is extremely important to identify new factors or interactors controlling or regulating PARP1 activity that can be therapeutic targets. Theoretically, to increase efficiency of PARP1 inhibitors in cancer therapy, we could modulate the activity of proteins related to its function; this could enhance clinical utility of PARP inhibitors and give further clue to address the resistance to PARP inhibitors. Moreover, the discovery of different interactors could lead to a more and efficient personalized therapy that may reduce development of resistance and expand therapy application of these drugs.

Results from our wide-genome screening in yeast showed that deletion of genes identified suppresses the growth inhibition phenotype caused by *PARP1* expression, suggesting that their activity is required to PARP1 to perform its function [31]. Moreover, cancers carrying mutations in these genes could be more sensitive to PARP1 inhibition, because our data suggest that their activity could efficiently modulate PARP1 activity. Therefore, this computational and integration analysis was aimed to find human genes (homologous to those ones identified in yeast) involved in pathways related to PARP1. Finally, we selected 12 genes that belong to cellular pathways chromatin remodeling and mRNA processing; it is known that PARP1 has a role in these cellular processes indicating that all those genes could be potential PARP1 functional interactors. Carcinogenesis often depends on mis-regulations of multiple pathways [46, 47], suggesting that related pathways function cooperatively and that their alteration could lead to a pathologic condition. On the other hand, the correlation of expression patterns could imply that these genes are involved in connected biological processes. Finally, combining functional and genetic information retrieved by several databases, we selected genes that are correlated to cancer development and also linked to PARP1. Among the 12 candidates, we decided to focus our analysis to those genes that genetically and functionally better correlate with PARP1 INCENP, RIT1 and PSAT1 resulted the most interesting candidates. In this study, we have also provided new evidence demonstrating that INCENP, RIT1 and PSAT1 may have a functional role in modulating PARP1 activity. We found that RIT1 and INCENP affect PARylation and PARP1 level, particularly in olaparib-treated cells; more interestingly, INCENP, RIT1 and PSAT1 have an influence on olaparib sensitivity in MCF7 cells. Altogether, these results suggest that INCENP, RIT1 and PSAT1 could affect PARP1 activity and are potentially candidates as therapy target to increase the efficacy of PARP1 inhibitors. Therefore, further investigation to better characterize the effect of these genes with PARP inhibitors could give interesting results for clinical therapy.

Deficiency in BER pathways induces genetic instability resulting in dramatic changes in gene expression and energy metabolism resembling changes found in many cancers [48]. Serine biosynthesis represents one of these pathways subjected to dramatic changes in cancer [49]. Moreover, BER deficient cells displayed a significant increase in expression of PSAT1, one of the key enzymes involved in serine biosynthesis, leading to an anabolic cellular state and an increased antioxidant capacity [50]. Thus, in cancer cells, PARP1 and PSAT1 inhibition could fail to promote changing in gene expression leading to cell death. Moreover, PSAT1 physically interacts with TALDO1, another candidate interactor of PARP1; therefore, their combined inhibition could improve PARP1-inhibitor efficiency and overcome resistance development [51].

Among all genes analyzed, INCENP has the highest expression correlation with PARP1, strongly indicating a functional relation between the two genes. In addition, our results showed a cooperative effect between PARP inhibition with olaparib treatment either on PARylation reduction or cell viability. These data suggest that, not only INCENP functionally interacts with PARP1, but also that this new function could be exploited to push cells to death.

In addition, our analysis identified NTRK1 kinase as a physical PARP1 interactor and, also, as a protein related to RIT1 pathway. This is particularly interesting because NTRK1 is a membrane-bound receptor that phosphorylates itself and members of the MAPK pathway [52] and it is already known that it confers resistance to cancer chemotherapy by activating p38 mitogen-activated protein kinase signaling pathways [53, 54], where RIT1 belongs. Our data support the idea that inhibition of RIT1 and PARP1 could lead to an excess DNA damage accumulation and, finally, to cell death. Therefore, it could be very informative to study the possible cooperation between PARP inhibitors and the RIT1 drugs we found by this analysis.

In conclusion, INCENP, RIT1 and PSAT1 seem to be the most interesting functionally-related genes that may modulate PARP1 activity. For these reasons, it should be important to check whether these genes carry mutations in tumors that are not responsive to PARP1 inhibitors.

Computational analysis and further functional validation in human cells demonstrated that “functional interactors” found by using yeast genome-wide screenings are likely to be relevant in human and may contribute to identify new molecular factors affecting disease progression and therapy. Yeast has been proven to be very useful to identify human proteins involved in HR and BRCA1-tumorigenesis [55, 56]. Recently, yeast-based screenings have been developed to identify new drug-combination for breast cancer therapy and new cancer associated genes [57, 58]. Our work confirms again the power of yeast genetics as tool to study functional interactions between human proteins owing to the conservation between this simple organism and humans.

## MATERIALS AND METHODS

### Database analysis and bioinformatics tools

To analyze the data set of 90 genes, we developed a script for R frame work to perform a pipeline for filtering and processing data from different sources; the steps are:

- data mining analysis on COSMIC and DisGeNET database;
- functional profiling using the “g:Profiler” web-based toolset;
- discovering for drug-gene interactions on DGIdb;
- data retrieving of all publications from NCBI-PubMed about the filtered dataset;
- gene alteration association and co-expression correlation analysis on cBioPortal database;
- interaction network analysis with GeneMANIA.

### Somatic mutation analysis

Information about the candidate genes identified with yeast screening such as characteristics and abundance of somatic mutations and distribution along different cancers has been taken from COSMIC database [59]. There are two types of data in COSMIC: expert curation data that are manually imputed from peer reviewed publications, and genome-wide screen data that are uploaded from publications reporting large scale genome screening data or imported from other databases such as TCGA (https://cancergenome.nih.gov/) and ICGC (http://icgc.org/icgc). Filtering our results for mutations found in cancer where PARP1 inhibition is already in use and where clinical trials are ongoing, we got the information about the number of variants for each gene present in database and relation to pathogenicity and cancer type where the gene is more often found mutated.

We also applied the 20/20 rule from Vogelstein et al. [44, 60] to divide these genes between “probable oncogenes” and “probable tumor suppressors”.

### Disease association analysis

Candidate genes have been analyzed also by the DIsGeNET (http://www.disgenet.org) that integrates human gene-disease associations (GDAs) from various expert curated databases and text-mining derived associations including Mendelian, complex and environmental diseases [61]. We used “disgenet2r”, an R package, enabling the development of bioinformatic workflow to query and analyze DisGeNET data, and visualize the results within R framework. We used the most recent released DisGeNET v5.0 (May, 2017). Data in DisGeNET are divided in curated and animal models; the first set of data is from databases such as UniProt, PsyGeNET, ORphanet, the second set from MGD (Mouse Genome Database) and RGD (Rat Genome Database). Gene-disease correlation was analyzed and a final score was given according to their level of evidence; this score ranges from 0 to 1 and takes into account the number and type of sources (level of curation, model organisms), and the number of publications supporting the association.

### Functional analysis

Functional enrichment analysis was performed by g:Profiler toolkit that studies multiple sources of functional evidence, including gene ontology terms, biological pathways, regulatory motifs of transcription factors and microRNAs, human disease annotations and protein-protein interactions (http://biit.cs.ut.ee/gprofiler/) [62].

### Drug-gene interactions and publications data retrieving

Information about drug-gene interactions for selected candidate genes has been retrieved with DGIdb (http://www.dgidb.org/search_interactions) by using the DGIdb API (Application Program Interface) through a simple JSON based interface [63]. For the identified genes, information has been retrieved from CIViC and Drugbank database with this algorithm. CIViC is an expert-crowdsourced knowledgebase for Clinical Interpretation of Variants in Cancer describing the therapeutic, prognostic, diagnostic and predisposing relevance of inherited and somatic variants of all types. Drugbank database is a bioinformatics and cheminformatics resource that combines detailed drug data with comprehensive target information [64, 65].

To retrieve publications from NCBI-PubMed, we used the R package RISmed that is an API for the Entrez Programming Utilities that provide a stable interface into the “Entrez” query and database system at NCBI. “Entrez” system currently includes many databases covering a variety of biomedical data, including nucleotide and protein sequences, gene records, three-dimensional molecular structures, and the biomedical literature [66].

### Alteration association and expression analysis

We analyzed correlation between alteration and expression of candidate genes and PARP1 in 8 different manual selected cancer data sets by taking advantage of cBioPortal database that provides visualization, analysis and download of large-scale cancer genomics data sets [67, 68]. We selected patients/samples related to cancer treated with PARP inhibitors. In particular: pancreatic cancer, uterine corpus endometrial carcinoma, ovarian serous cystadenocarcinoma, neuroendocrine prostate cancer, breast invasive carcinoma, liver hepatocellular carcinoma, stomach adenocarcinoma and sarcoma. After this selection, we performed a statistical mutual exclusivity or co-occurrence analysis between alterations in our candidate genes and PARP1; copy number alterations, amplifications, deletions and mutations were analyzed. Results are shown as *p*-value of frequency of this association and a statistical correlation has been discovered only for gene amplification. Then, we performed a co-expression analysis to see if the expression of our candidate genes is related to expression of PARP1. Results are expressed as “Pearson correlation” coefficient (p). 0 < p < 0.3 means weak correlation, 0.3 < p < 0.6 medium correlation and p > 0.6 high correlation.

### Interaction network

Interaction network analysis has been performed with GeneMANIA [69] ( https://genemania.org/) that finds interconnections between proteins in term of co-expression, physical interaction, genetic interaction, shared protein domains, co-localization and common pathway. Two different analyses have been made: first, it has been studied the interaction network just between the 12 proteins identified from DisGeNET and then, a deeper analysis on the most promising proteins retrieved from cBioPortal to include also secondary members of the “interaction” network.

### Expression analysis

Differential expression analysis related to INCENP, RIT1 and PSAT1 has been analyzed in “EMBL-EBI Expression Atlas” (https://www.ebi.ac.uk/gxa/home) that gives information on the abundance and localization of RNA (and proteins) across species under different biological conditions [70, 71]. As triple negative breast and high ovarian serous cancers are reported to be currently treated with PARP inhibitors [72] [73], we analyzed and compared RIT1-, INCENP- and PSAT1-expression in hormonal receptors (triple) negative breast cancer cell lines and hormonal receptors positive (+) breast cancer cell lines, and in high ovarian serous cancer cell lines and clear cell ovarian cancer cell lines. Data were statistically analyzed by using the Student's *t*-test for unpaired data. List of cell lines analyzed and genes expression data are showed in Supplementary Table 5.

### Cell transfection, drug treatments, qRT-PCR and western blot

MCF7 breast cancer cell line was used for cellular validation experiments. MCF7 cells were cultured in Dulbecco's Modified Eagle's Medium (DMEM) high-glucose with 10% fetal bovine serum with the addition of sodium pyruvate and not essential amino-acids (NEA).

Olaparib was obtained from Selleckchem; stock solutions were made in DMSO at 10, 5, 2, 1 and 0.5 mM and stored at −20°C in the dark. Stock solutions were diluted 1/1000 directly in culture medium to reach the final concentration.

SiRNAs against selected genes (PSAT1, RIT1 and INCENP) were transfected by using the “Lipofectamine 2000” transfection protocol as recommended by the manufacturer (ThermoFisher). After 48 hours, total RNA was extracted with QIAzol lysis reagent protocol (Qiagen). Amounts corresponding to 1μg of RNA have been retro-transcribed to cDNA and analysed by qRT-PCR to evaluate the expression level of RIT1, PSAT1 and INCENP. Glyceraldehyde-3-Phosphate Dehydrogenase (GAPDH), phorphobilinogen deaminase (PBDG) and succinate dehydrogenase (SDHA) have been used as housekeeping genes. Sequence of siRNA and primers are available upon request.

Protein extracts from olabarip-treated and/or siRNA transfected cells (48 hours after transfection) were performed by re-suspending cells in lysis buffer on ice for 30 minutes, then cell suspensions were sonicated for 25 minutes and centrifuged at 14000g at 4°C for 30 minutes. Supernatants with total proteins were recovered and quantified. Western blots were carried out as follows: 30μg of total cell were loaded on 10% polyacrylamide pre-casted gel (Invitrogen) and analyzed by SDS-PAGE. Thereafter, proteins were transferred to nitrocellulose membrane for antibodies hybridization. Membrane was blocked with 5% milk; all primary antibodies were incubated at 4°C overnight and anti-mouse and anti-rabbit secondary antibodies were incubated for 1 hour at room temperature. Anti-PAR rabbit polyclonal antibody (4336-BPC-100) was purchased from Trevigen, anti-PARP mouse monoclonal antibody (sc-8007) and mouse monoclonal anti-INCENP (sc-376514) from Santa Cruz Biotechnology, anti-RIT1 rabbit polyclonal antibody (PA5-30919) and anti-PSAT1 rabbit polyclonal antibody (PA5-22124) from ThermoFisher Scientific. Densitometry was performed by computer assistance; results are the mean of 3–4 independent gels ±SD. To determine if RIT1, INCENP and PSTA1 affects olaparib sensitivity MCF 7 cells were seeded in 6 wells at 90% confluency and transfected with for siRNA as described before. After 4-6 hours of transfection, 6×10^3^ cells were seeded in 12-wells plates, treated in duplicate with DMSO and five different concentration of olaparib (0.5, 1, 2, 5 and 10 μM). After 48 hours, cells were fixed with paraformaldehyde at 4% for 10 minutes, stained with crystal-violet for 15 minutes and washed. The day after, cells were detached with acetic acid and counted. Each line with different siRNA was normalized with its DMSO and for each concentration. Results are the mean of 3–4 independent experiments ± SD. Statistical analysis was carried out by Student's *t*-test.

### Availability of data and materials

All the data and materials used in this study are available at Institute of Clinical Physiology, CNR, Pisa, Italy.

## SUPPLEMENTARY MATERIALS TABLES











## Abbreviations

PARP1: poly (ADP-ribose) polymerase 1
SSB: single strand breaks
DSB: double strand breaks
HR: homologous recombination
NHEJ: non-homologous end joining
COSMIC: catalogue of somatic mutations in cancer
BER: base excision repair
NER: nucleotide excision repair
DisGeNET: database of gene-disease associations
DGIdb: Drug Gene Interaction Database
GAD: Genetic Association Database
LHGDN: Literature-derived Human Gene-Disease Network
MGD: Mouse Genome Database
RGD: Rat Genome Database
CIViC: Clinical Interpretations of Variants In Cancer
DMEM: Dulbecco’s Modified Essential Medium
NEA: Non Essential Aminoacids
GAPDH: Glyceraldehyde-3-Phosphate Dehydrogenase
PBDG: phorphobilinogen deaminase
SDHA: succinate dehydrogenase
