# Supplementary material for: Computational analysis of data from a genome-wide screening identifies new PARP1 functional interactors as potential therapeutic targets
Source: Oncotarget. 2019 Apr 12;10(28):2722–37. doi: 10.18632/oncotarget.26812 (PMC6505629; doi:10.18632/oncotarget.26812)
Supplement: Supplementary file 1 [file oncotarget-10-2722-s001.pdf]

Computational analysis of data from a genome-wide screening identifies new *PARP1* functional interactors as potential therapeutic targets

SUPPLEMENTARY MATERIALS

Supplementary Table 1: Identification of oncogenes and tumor soppressors by applying the 20/20 rule.

See Supplementary File 1

Supplementary Table 2: The identified genes are found associated with different types of cancer. Here, information about cancer type, gene description and relevant publications is reported.

See Supplementary File 2

Supplementary Table 3: Functional analysis showed that PARP1 is related to all the identified genes.

See Supplementary File 3

Supplementary Table 4: Expression correlation values expressed as Pearson coefficient (p), cancer and genes analyzed are reported

CANCER	CANDIDATE GENE											
	RIT1	HERC3	ENTPD6	NCOR1	RCOR1	H1F0	INCENP	PSAT1	REXO1	TALDO1	ZFP36	ZFP36L2
breast	0.26	-0.14	-0.02	0.04	0.04	0.03	0.33	0.1	-0.12	-0.02	-0.18	-0.12
liver	0.27	-0.03	0.1	0.12	0.04	-0.06	0.3	0.02	0.18	0.01	-0.16	-0.05
ovarian	0.03	-0.14	-0.09	0.12	0.12	0.03	0.24	0.1	-0.05	-0.05	-0.19	0.01
prostate	0.45	0.58	0.31	0.17	0.46	0.56	0.63	0.34	0.35	0.36	-0.16	-0.14
sarcoma	0.19	-0.13	-0.13	0.04	0.1	0.29	0.27	0.05	0.11	-0.09	-0.18	-0.18
stomach	0.14	-0.04	0.07	0.08	0.23	-0.09	0.45	0.23	0.25	0.06	-0.22	0.02
uterine	0.26	0.06	-0.15	0.18	0.3	0.09	0.36	0.1	-0.27	-0.04	-0.12	-0.04

$0 < p < 0.3$ weak direct correlation
$0.3 \le p < 0.6$ medium direct correlation
$0.6 \le p < 0.9$ high direct correlation
$-0.3 < p < 0$ weak inverse correlation
$-0.6 < p \le -0.3$ medium inverse correlation
$-0.9 < p \le -0.6$ high inverse correlation

Supplementary Table 5: Expression analysis with "expression atlas" database of selected genes, in cell lines representing cancer sensitive to PARP inhibition and not. For breast cancer: double/triple negative breast cancer cell lines as sensitive and normal cancer as not sensitive, for ovarian cancer: high grade serous as sensitive and clear cancer cell as not sensitive. Data are shown as transcript per million (TPM) from different sources and a t-test for unpaired data has been performed between means of expression in the two different models.

See Supplementary File 4
